# Speech and swallowing characteristics in patients with facioscapulohumeral muscular dystrophy

**DOI:** 10.1590/0004-282X-ANP-2021-0034

**Published:** 2022-02-21

**Authors:** Vanessa Brzoskowski dos SANTOS, Jonas Alex Morales SAUTE, Laís Alves JACINTO-SCUDEIRO, Annelise AYRES, Rafaela Soares RECH, Alcyr Alves de OLIVEIRA, Maira Rozenfeld OLCHIK

**Affiliations:** 1 Universidade Federal de Ciências da Saúde de Porto Alegre, Programa de Pós-Graduação em Ciências da Reabilitação, Porto Alegre RS, Brazil.; 2 Universidade Federal do Rio Grande do Sul, Departamento de Medicina Interna, Porto Alegre RS, Brazil.; 3 Universidade Federal do Rio Grande do Sul, Porto Alegre RS, Brazil.; 4 Universidade Federal de Ciências da Saúde de Porto Alegre, Porto Alegre RS, Brazil.; 5 Universidade Federal do Rio Grande do Sul, Departamento de Cirurgia e Ortopedia, Porto Alegre RS, Brazil.

**Keywords:** Muscular Dystrophy, Facioscapulohumeral, Dysarthria, Speech, Deglutition Disorders, Neuromuscular Diseases, Distrofia Muscular Facioescapuloumeral, Disartria, Fala, Transtornos de Deglutição, Doenças Neuromusculares

## Abstract

**Background:**

Although facial muscle weakness is common in patients with Facioscapulohumeral Muscular Dystrophy (FSHD), the literature is scarce on the speech and swallowing aspects.

**Objective:**

To investigate speech and swallowing patterns in FSHD and assess the correlation with clinical data.

**Methods:**

A cross-sectional study was conducted. Patients with clinical confirmation of FSHD and aged above 18 years were included and paired with healthy control individuals by age and gender. Individuals who had neurological conditions that could interfere with test results were excluded. The following assessments were applied: speech tests (acoustic and auditory-perceptual analysis); swallowing tests with the Northwestern Dysphagia Patient Check Sheet (NDPCS), the Eat Assessment Tool (EAT-10), the Speech Therapy Protocol for Dysphagia Risk (PARD), and the Functional Oral Intake Scale (FOIS); disease staging using the modified Gardner-Medwin-Walton scale (GMWS); and quality of life with the Medical Outcomes Study 36-Item Short-Form Health Survey (SF-36). The correlation between test results and clinical data was verified by non-parametric statistics.

**Results:**

Thirteen individuals with FSHD and 10 healthy controls were evaluated. The groups presented significant differences in the motor bases of phonation and breathing. Regarding swallowing, two (15%) individuals presented mild dysphagia and seven (53.8%) showed reduced facial muscles strength. These results were not correlated with duration of the disease, age at symptoms onset, and quality of life. Dysphagia was related to worsening disease severity.

**Conclusions:**

FSHD patients presented mild dysarthria and dysphagia. Frequent monitoring of these symptoms could be an important way to provide early rehabilitation and better quality of life.

## RESUMO

**Antecedentes:**

Embora haja predomínio de fraqueza muscular facial na distrofia facioescapuloumeral (FSHD), é escassa a literatura sobre aspectos de fala e deglutição.

**Objetivo:**

Investigar os padrões de fala e deglutição na FSHD e correlacioná-los com dados clínicos da doença.

**Métodos:**

Estudo transversal. Pacientes com confirmação clínica de FSHD e idade acima de 18 anos foram incluídos e pareados por idade e sexo com controles saudáveis. Foram excluídos indivíduos que apresentassem condições neurológicas que pudessem interferir nos resultados dos testes. Aplicaram-se as seguintes avaliações: fala (análise acústica e perceptivo-auditiva); deglutição, por meio do *Northwestern Dysphagia Patient Check Sheet* (NDPCS), *Eat Assessment Tool* (EAT-10), Protocolo de Avaliação para Risco de Disfagia (PARD) e *Functional Oral Intake Scale* (FOIS); estadiamento da doença, por meio da *Gardner-Medwin-Walton scale* (GMWS); e qualidade de vida, com o *Medical Outcomes Study 36-Item Short-Form Health Survey* (SF-36). Resultados de fala e deglutição foram correlacionados com dados clínicos da doença por teste não paramétrico.

**Resultados:**

Foram avaliados 13 indivíduos com FSHD e dez controles saudáveis. Houve diferença significativa entre os grupos nas bases motoras fonação e respiração. Na deglutição, dois (15%) indivíduos apresentaram disfagia leve e sete (53,8%), força reduzida da musculatura da face. Esses resultados não foram correlacionados com tempo de doença, idade de início dos sintomas e qualidade de vida. A disfagia esteve relacionada com a gravidade da doença.

**Conclusões:**

Pacientes com FSHD apresentaram disartria e disfagia leves. O monitoramento frequente desses sintomas pode ser uma forma importante de proporcionar reabilitação precoce e melhor qualidade de vida.

## INTRODUCTION

Facioscapulohumeral Muscular Dystrophy (FSHD) is a genetic neuromuscular disease characterized by muscle weakness and progressive atrophy^1^ ^,^ ^2^. FSHD is one of the most frequent forms of muscular dystrophy in adults, with an estimated prevalence between four and ten per 100,000 population^3^. This disease primarily affects the facial muscles, scapula muscles, and humerus muscles^4^. One of the classical symptoms is weakness of the facial muscles, which is present in 80% of patients with FSHD, with the orbicularis and the greater zygomatic muscles being the most affected^5^ ^,^ ^6^. Although face muscular weakness is common in these individuals, the literature is scarce with regard to the speech (dysarthria) and swallowing (dysphagia) aspects.

Dysarthria is a speech disorder resulting from disturbances in neuromuscular control of speech mechanisms, which may compromise the functions of breathing, phonation, resonance, articulation, and prosody^7^. In a recent study, decreasing strength of facial muscles was found to be related to communication difficulties in patients with FSHD^8^. Despite the speech difficulty noted by these patients, very little is known about the characteristics of their dysarthria in FSHD, and its correlation with the clinical profile of the disease.

Dysphagia is a swallowing disorder caused by neurological disease and/or an obstruction that causes difficulty in safe deglutition from the mouth to the esophagus^9^. The literature about dysphagia in FSHD is also scare and most relates to its incidence. The prevalence of dysphagia in FSHD ranges from 2 to 25%, and it is usually characterized by a mild dysphagia that occurs in advanced stages^10^ ^,^ ^11^ ^,^ ^12^ ^,^ ^13^. In a study conducted with eight FSHD patients, six of them had mild dysphagia with fragmented swallowing and weakness of the tongue and jaw muscles^11^.

AS there are no publications in the literature on the standardized, objective and detailed characterization of speech and swallowing aspects in FSHD, further studies in this area are needed to better understand the disease, and support the early rehabilitation of these individuals. Thus, the aim of this study was to characterize speech and swallowing patterns in patients with FSHD, and assess their correlation with clinical data on the severity of the disease.

## METHODS

### Study design and population

This was a cross-sectional study conducted at a neuromuscular genetic disease care center within a hospital in Porto Alegre, in the southern region of Brazil, from April to November 2019. Unrelated and healthy controls, matched for age and sex, were recruited from the community.

The inclusion criteria were:

- Clinical diagnosis of FSHD.
- Aged 18 years or over.

The exclusion criteria were:

- Presence of other neurological or systemic conditions that can impact speech and swallowing patterns (for example, head and neck tumor).
- Unsuccessful attempt at telephone contact.
- Individuals who did not show up for the scheduled appointment.
- Patients who refused to participate in the study.

Initially, 26 patients with FSHD were recruited from the database of the care center at the hospital, 13 of which were excluded for the following reasons: five due to contact failure, four due to missing scheduled appointment, three refused to participate in the study, and one was under 18 years of age. The final sample of the study comprised 13 individuals (seven families) with FSHD.

The project was approved by the hospital’s Research Ethics Committee. All participants gave their written consent before participating in the study.

### Data collection

Individual with FSHD underwent assessments and answered the following questionnaires:

- *Sociodemographic questionnaire*: a structured questionnaire to collect general patient data, such as age, sex, education level, age of onset of symptoms, and duration of illness.
- *Gardner-Medwin-Walton* (GMWS): a clinical scale to quantify the neurological severity of FSHD. The instrument is divided into 10 (0-9) increasing severity levels^14^.
- *Medical Outcomes Study 36-Item Short-Form Health Survey* (SF-36): a quality of life assessment instrument consisting of 8 dimensions: physical function, role-physical, bodily pain, general health, vitality, social function, role-emotional and mental health. Each dimension can be scored between 0 to 100 with higher scores indicating better health^15^.
- *Mini-Mental State Examination (MMSE):* screening test translated and validated for the Brazilian population. The cutoff used for formal education is 28 points for more than 8 years; 26 points for 5 and 8 years; 25 points for 1 and 4 years; and 20 points for illiterate^16^.

### Speech assessment

- *Speech assessment:* this evaluation involves tasks to test the five subsystems of speech: phonation (sustaining the vowel /a/ in a single breath), resonance (­sustaining the vowel /a/ in a single breath), prosody (counting from 20 to 30); respiration (sustaining the vowel /a/ in a single breath), and articulation (alternating the sequence of syllables [pataka] as fast as personal capacity allowed, repeatedly in a single breath; alternating the sequence I-U [i:ju:], repeatedly in a single breath*).* The Audacity software version 2.3.2 was used to record patients in a soundproof environment with a KARSECT HT-9 microphone and an Andrea Pureaudio USB adapter positioned approximately 5 cm from the subject’s lips.
- *Perceptual-auditory speech analysis:* for the auditory-perceptual analysis, five speech therapists blinded to the patients’ diagnosis analyzed the recordings and classified each of the five speech subsystems as normal or altered (mild, moderate or severe). The speech therapists were trained and had a Kappa concordance coefficient greater than 0.90.
- *Speech acoustic analysis:* the PRAAT 5.1 software (www.praat.org) was developed by linguists Paul Boersma and David Weenink and its focus is sound analysis through parameters such as frequency, wavelength, decibels, among others^17^. The representation of these aspects, normative values, and corresponding speech tasks are described in the Table 1.

**Table 1.** Acoustic evaluation: motor bases, tasks performed, and outcomes.

Motor base	Assignment	Resulting variable
Phonation	Sustaining the A vowel in a single breath.	Fundamental frequency (Fo): For Brazilian Portuguese speakers, the frequency range of normality for females is 150-250 Hz and 80-150 Hz for males^18^. Jitter rap: The normative values of PRAAT is 0.680% as a threshold for pathology for *jitter rap* ^17^. Shimmer local: The normative values of PRAAT is 3.810% as a threshold for pathology for *shimmer local* ^17^.
Resonance	Sustaining the A vowel in a single breath.	Extraction of the third and fourth formants of sustained vowel A.
Prosody	Counting from 20 to 30	Fundamental frequency of count: maximum fundamental frequency (Fo max), minimum fundamental frequency (Fo min), and standard deviation of fundamental frequency.
Breathing	Sustaining the A vowel in a single breath.	Maximum phonation time (MPT): For Brazilian Portuguese speakers, the standard of normality for females is 14 seconds and for males, 20 seconds^18^.
Articulation	Alternating repetition of [pataka] the fastest in a single breath. Repetition diphthong I-U [i:ju:] alternately in a single breath.	Diadochokinesis (DDK): Young adults 6.58 syllables per second and elderly people, 6.13 syllables per second^19^. Extraction of the first and the second formants of repetition of two combined vowels.

### Swallowing assessment

A drink of water (100 mL) was offered during the functional test and the following instruments were applied:

- *Northwestern Dysphagia Patient Check Sheet* (NDPCS): comprises a brief clinical and functional evaluation of swallowing consisting of 28 items divided into three parts: medical history and behavioral variables, gross motor function, and an oral motor test^20^.
- *Eat Assessment Tool* (EAT-10): evaluates the emotional impact and physical symptoms that swallowing problems may have on the individual’s life, with a score ranging from 0 to 40; scores greater than 3 indicate a risk for oropharyngeal dysphagia^21^.
- *Speech-Language Pathology Assessment for Dysphagia Risk* (PARD): used to classify normality, mild dysphagia, mild to moderate dysphagia, moderate dysphagia, moderate to severe dysphagia, and severe dysphagia^22^.
- *Intake Scale* (FOIS): ranges from zero to seven, with a score of zero indicating that no oral diet is recommended and a score of seven indicating that a normal oral diet is recommended without restrictions^23^.

### Statistical analysis

The independent variables, the perceptual speech analysis, and the swallowing evaluation were presented through descriptive analyzes (absolute and relative frequencies and mean and standard deviation or median and interquartile range). The statistical test was selected according to the data distribution provided by the Shapiro-Wilk test and histograms. For the acoustic analysis of speech between groups, the Mann-Whitney test was used and for the correlation of speech scores with independent variables, the Spearman correlation test was used. Statistical significance was defined as p<0.05. The statistical software used was SPSS version 22.0.

## RESULTS

Thirteen individuals (seven families) with FSHD and 10 healthy controls were enrolled. The mean duration of disease in the FSHD group was 6.7 (SD=5.9) years and six (46.1%) individuals in this group showed a neurological severity of 4 in the GMWS scale. The FSHD group presented lower scores of quality of life in the dimensions of physical function, role-physical, and bodily pain than the control group. Table 2 shows the clinical and demographic characteristics of individuals with FSHD and controls.

**Table 2.** Demographic data of the FSHD and control groups.

		FSHD (n=13)	Controls 1 (n=10)	p-value
Female		9 (69.2%)	6 (60%)	0.663
Age		49.5 (13.2)	45.6 (12.2)	0.474
Educational level (years)		8.6 (4.1)	-	
Age of disease onset		42.7 (15.9)	-	
Disease duration		6.7 (5.9)	-	
GMWS - severity level	Normal - 0	1 (7.7%)	-	
	1	2 (15.4%)		
	2	0 (0%)		
	3	0 (0%)		
	4	6 (46.1%)		
	5	2 (15.4%)		
	6	1 (7.7%)		
	7	0 (0%)		
	8	1 (7.7%)		
	Severe - 9	0 (0%)		
MMSE	Normal	6 (46.1%)		
	Altered	7 (53.9%)		
SF-36	Physical Function	30 (7.5-45.0)		
	Role-Physical	0 (0-62.5)		
	Bodily Pain	21 (15-46)		
	General Health	27 (26-48.5)		
	Vitality	60 (25-65)		
	Social Function	50 (31.2-75)		
	Role-Emotional	33.50 (0-83.2)		
	Mental Health	76 (42-89)		

Data are reported means (standard deviation), except for sex, MMSE, and GMWS scores which are reported as frequency. SF-36 data are reported as medians (interquartile range). FSHD: Facioscapulohumeral muscular dystrophy; GMWS: Gardner-Medwin and Walton Scale; MMSE: Mini Mental State Examination; SF-36: Medical Outcomes Study 36-Item Short-Form Health Survey.

### Speech results

Auditory-perceptual analysis of the subsystems of speech of the FSHD and control groups are shown in Table 3. A statistical difference was found between the FSHD and control groups regarding the phonation and respiration subsystems (Table 4). The speech data from the acoustic analysis did not show a significant correlation with GMWS scores, duration of the disease, age at onset of symptoms, or the quality of life of these individuals.

**Table 3.** Auditory-perceptual analysis.

	FSHD		Controls	
	(n=13)	Classification	(n=10)	Classification
Phonation	6 (46.2%)	Mild impairment	1 (7.7%)	Mild impairment
Respiration	1 (7.7%)	Mild impairment	0 (0%)	-
Resonance	1 (7.7%)	Mild impairment	0 (0%)	-
Articulation	3 (23.1%)	Mild impairment	0 (0%)	-
Prosody	0 (0%)	-	0 (0%)	-

Data are expressed as frequency. FSHD: Facioscapulohumeral muscular dystrophy.

**Table 4.** Comparison of acoustic parameters between groups.

	FSHD (n=13)	Controls 1 (n=10)	p-value
Jitter (local)	0.70% (0.44-1.23)	0.33% (0.23-0.75)	0.044*
Jitter (rap)	0.36% (0.21-0.65)	0.18% (0.11-0.39)	0.032*
Shimmer (local)	10.79dB (8.72-14.39)	7.90dB (4.72-10.43)	0.030*
FF average vowel	161.02Hz (127.23-191.91)	162.03Hz (111.55-207.98)	0.951
FF minimum vowel	125.12Hz (93.35-168.20)	94.64Hz (76.92-155.36)	0.264
FF maximum vowel	251.18Hz (171.20-403.76)	215.16Hz (129.90-411.85)	0.804
FF SD vowel	18.86Hz (3.50-59.85)	20.38Hz (1.11-47.56)	0.951
MPT vowel^a^	6.09 (3.61-9.33)	9.37 (8.28-12.25)	0.047*
FF average counting	187.96Hz (144.01-216.71)	168.28Hz (99.62-186.10)	0.094
FF minimum counting	77.13Hz (73.69-92.82)	80.24Hz (77.24-85.89)	0.620
FF maximum counting	442.60Hz (348.69-487.03)	445.17Hz (253.37-491.90)	0.710
FF SD counting	70.45Hz (39.96-84.77)	39.17Hz (20.93-58.32)	0.063
PATAKA^a^	5.59 (4.73-6.07)	5.62 (4.61-6.45)	0.852
IU^a^	0.88 (0.81-0.98)	0.95 (0.83-1.24)	0.336
IU F1	523.04Hz (457.17-570.53)	510.80Hz (470.88-622.22)	0.598
IU F2	1752.25Hz (1671.29-1823.87)	1836.56Hz (1614.20-1904.91)	0.251
IU F3	2964.16Hz (2807.62-3095.68)	2983.76Hz (2856.18-3038.25)	0.687
IU F4	4032.51Hz (3939.74-4112.93)	3926.34Hz (3833.21-4030.95)	0.114

Data are reported as medians (interquartile range); *p<0.05; FSHD: Facioscapulohumeral muscular dystrophy; FF: Fundamental Frequency; MPT: maximum phonation time; ^a^syllables per second.

Due to sex difference in fundamental frequency of speech, the acoustic analysis was performed between groups of females only. Only the respiration subsystem showed a significant difference between female FSHD and control participants [5.95 (3.72-7.50) and 9.37 (8.46-11.45), p=0.009]. The speech acoustic in males was not performed because of the small sample size (there were only four male participants with FSHD). This result did not have a significant correlation with the clinical data (Table 5).

**Table 5.** Correlations between speech disorders, clinical variables, and quality of life.

	Jitter (local)		Jitter (rap)		Shimmer (local)		MPT	
	p-value	r	p-value	r	p-value	r	p-value	r
Initial symptoms	0.127	-	0.066	-	0.072	-	0.129	-
Length of illness	0.128	-	0.249	-	0.053	-	0.868	-
GMWS	0.406	-	0.738	-	0.603	-	0.334	-
SF-36 Physical Function	0.665	-	0.411	-	0.485	-	0.559	-
SF-36 Role-Physical	0.983	-	0.657	-	0.622	-	0.991	-
SF-36 Bodily Pain	0.114	-	0.073	-	0.110	-	0.355	-
SF-36 General Health	0.053	-	0.053	-	0.151	-	0.437	-
SF-36 Vitality	0.872	-	0.843	-	0.760	-	0.914	-
SF-36 Social Function	0.408	-	0.188	-	0.636	-	0.328	-
SF-36 Role-Emotional	0.199	-	0.107	-	0.092	-	0.668	-
SF-36 Mental Health	0.964	-	0.552	-	0.324	-	0.205	-

MPT: maximum phonation time; GMWS: Gardner-Medwin and Walton Scale; SF-36: Medical Outcomes Study 36-Item Short-Form Health Survey.

### Swallowing results

NDPCS results were verified to identify items that showed the greatest changes. Seven (53.8%) patients presented altered oral muscle tone while nine (69.2%) demonstrated no pharyngeal contraction of the gag reflex. Also, six (46.1%) presented deviated lip protrusion and nine (69.2%) reported not being able to move their lips, such as in pouting and whistling. Of the 13 patients with FSHD, two (15%) were diagnosed with mild dysphagia. The average EAT-10 score was 1.15 (±1.86). Regarding the functionality, 85% of the patients presented a FOIS 7 and 15% were a FOIS 6 (15%).

Regarding the NDPCS instrument, there was a positive correlation with the GMWS (r=0.604, p=0.029), showing that the greater the neurological severity the higher the score on this instrument. When the two patients who had a diagnosis of mild dysphagia were individually analyzed, we found that both scored the highest values in the GMWS (eight and six). There was no correlation between the swallowing data from the NDPCS instrument with other clinical data and with quality of life: initial symptoms (p=0.805); duration of illness (p=0.609); physical function (p=0.148); role-physical (p=0.186); bodily pain (p=0.869); general health (p=0.053); vitality (p=0.446); social function (p=0.753); role-emotional (p= 547); and mental health (p=0.250).

## DISCUSSION

In this study, we carried out a detailed characterization of speech and swallowing in FSHD patients undergoing treatment at a neuromuscular genetic disease care center in southern Brazil. Almost half of the patients were diagnosed with mild dysarthria after auditory-perceptual and acoustic analysis of their speech, with changes in the phonation and respiration subsystems. We found mild dysphagia in 15% of the patients, and the risk for dysphagia observed with the EAT-10 is compatible with swallowing findings.

Almost half of the patients with FSHD had alterations in the motor base of phonation in the auditory-perceptual analysis when compared to the control group. In the acoustic analysis including both sexes, alterations in the motor bases of phonation and breathing were observed, corroborating the findings in the auditory-perceptual analysis. This indicates the importance of carrying out both analyzes, as the acoustic analysis proved to be complementary to the auditory analysis^7^. Auditory-perceptual analysis assesses the global impression of vocal quality and is considered the gold standard of analysis^24^.

Regarding the motor base of breathing, measured in the acoustic analysis by the maximum phonation time (MPT), patients with FSHD presented values well below normal and lower than the control group^18^. One hypothesis is that this reduction in maximum phonation time may be related to muscle fatigue and weakness^8^ ^,^ ^25^ ^,^ ^26^. FSHD patients have respiratory muscle weakness as an intrinsic characteristic of the disease, which can involve the diaphragm and expiratory abdominal muscles^27^. Clinically, these individuals can have communication difficulties and fatigue with long speeches. Additionally, breathing is closely related to phonation as it affects the synchrony between aerodynamic and myoelastic mechanisms, leading to coordination disorders in affected individuals^28^.

In the phonation subsystem, six (46.2%) patients presented mild changes in the auditory-perceptual analysis. As in the acoustic analysis, patients with FSHD had significantly higher values for shimmer and jitter when compared to the control group. Changes in this motor base possibly also occur due to fatigue, muscle weakness, or breathing changes. These changes in vocal quality interfere with speech intelligibility and can have a critical impact on communication skills, which in turn limits the individual’s occupational, educational and social abilities. Correct characterization of these changes can allow a more specific and early rehabilitation^29^.

Even though FSHD patients had reduced strength in facial muscles, impaired articulation was observed in three (21.3%) patients. In the acoustic analysis, this motor subsystem was similar in patients and control groups. Diadochokinesis is widely used to measure the articulatory quality of neurological patients^30^. The initial hypothesis is that since this was a sample with a predominance of individuals with mild disease, joint damage may not yet have been apparent in this population.

In the swallowing evaluation, only 15% of the patients had mild oropharyngeal dysphagia, characterized by changes in the pharyngeal phase (delay in pharyngeal swallowing, reduction in pharyngeal elevation, and multiple swallowing). In a videofluoroscopy study, it was observed that the decrease in tongue strength in patients with FSHD is consistent with the delay in pharyngeal swallowing^11^. Changes in swallowing were related to worsening disease severity. These data are in line with the literature, where the reported prevalence of dysphagia in patients with FSHD ranges from 2 to 25%, being generally characterized by mild dysphagia and occurring in more advanced cases of the disease^8^ ^,^ ^10^ ^,^ ^11^ ^,^ ^12^ ^,^ ^13^.

The majority of patients had reduced strength of facial muscles and absence of the GAG reflex. These findings agree with those of other studies that mainly reported weakness of the tongue and jaw and decreased resistance to cheek compression^8^ ^,^ ^11^. Patients presented deviated lip protrusion and reported not being able to make certain lip movements (such as puckering and whistling) and this may be related to the orbicularis oris and zygomaticus major muscles, which are the most affected muscles in these patients^5^.

Regarding the eating function, 85% of the patients maintained a normal oral diet (FOIS 7) and 15% had some food restriction (FOIS 6). The self-assessment of the risk for dysphagia of patients with FSDH measured by the EAT-10 was compatible with swallowing findings, demonstrating reliability in patients’ self-perception, which may be useful in the early identification of symptoms.

It is important to note that there was no correlation between speech and swallowing changes with quality of life. This might indicate that the current mild changes might not impact quality of life. In addition, as changes were also unrelated to the duration of the disease and the age of onset of symptoms, they may not follow the course of the disease but occur at different times and require constant clinical attention to detect symptoms.

The main limitation of this work was the sample size and the lack of a molecular diagnosis of FSHD. Considering that previous reports suggest that FSHD-related D4Z4 contractions are responsible for 95% of FSHD cases, our results must be interpreted as mainly related to this subtype of the disease^3^. Another limitation of this work relates to the lack of objective assessment of swallowing in these patients. The above findings show that the topic is relevant and would be best investigated longitudinally, to identify at what point in the course of the disease these changes occur and how they vary in the course of the disease.

In conclusion, FSHD patients had mild dysarthria (with changes in the phonation and respiration subsystems) and mild dysphagia, which is associated with disease severity. Thus, the frequent monitoring of these symptoms by physicians may be important to ensure early rehabilitation and a better quality of life for these patients.
